# Subsurface aeration mitigates organic material mulching-induced anaerobic stress *via* regulating hormone signaling in *Phyllostachys praecox* roots

**DOI:** 10.3389/fpls.2023.1121604

**Published:** 2023-03-01

**Authors:** Jianshuang Gao, Shunyao Zhuang, Renyi Gui

**Affiliations:** ^1^ State Key Lab of Soil and Sustainable Agriculture, Institute of Soil Science, Chinese Academy of Sciences, Nanjing, China; ^2^ College of Modern Agricultural Sciences, University of Chinese Academy of Sciences, Beijing, China; ^3^ State Key Lab of Subtropical Silviculture, Zhejiang Agriculture & Forestry University, Hangzhou, China

**Keywords:** mulching, Aux/IAAs, hormones, anaerobic enzyme, soil aeration

## Abstract

Organic material mulching has been used extensively to allow *Phyllostachys praecox* to promote growth and development of shoots. However, the bamboo forest always showed a significant degradation, probably due to anaerobic damage caused by the mulching after several years. Therefore, we have innovatively proposed an improvement measure to aerate the underground pipes for the first time. We investigated the role of subsurface pipe aeration in regulating root hypoxia to reduce the stress and to identify the degradation mechanism. Results showed that aeration increased oxygen concentration, shoot yield and root growth compared with mulching, and the aeration enhanced the concentration of indole-3-acetic acid (IAA) and the expression of *Aux/IAAs* (*Aux1*, *Aux2*, *Aux3*, and *Aux4*). Aeration reduced gibberellin (GA), ethylene (ETH), and abscisic acid (ABA) contents as well as anaerobic enzyme activities (alanine transaminase, AlaAT; alcohol dehydrogenase, ADH; pyruvate decarboxylase, PDC; and lactate dehydrogenase, LDH), which alleviated root damage in anoxic conditions. Furthermore, correlation showed that the activities of ADH, LDH, PDC, and AlaAT showed significant linear correlations with soil oxygen levels. RDA analyses showed that ABA, IAA, and ETH were found as the key driving hormones of *Aux/IAAs* in the root of the forest mulched with organic material. Here we show that subsurface aeration increases soil oxygen concentration, shoot yield, root growth and regulates phytohormone concentrations and *Aux/IAAs* expression, which reduces anaerobic enzyme activities. Consequently, subsurface pipe aeration is an effective measure to mitigate the degradation of bamboo forests caused by soil hypoxia that results from organic material mulching.

## 1 Introduction

*Phyllostachys praecox* f. *preveynalis* is a bamboo species in the family Gramineae that is widespread in southern of China (Gui et al., 2013). The shoots of *P. praecox* are known as “the best bamboo shoots in China” because of their delicious taste, early season harvesting, high yield, and economic impact (Huang et al., 2007; Guo et al., 2015). The most effective way to obtain greater benefits is to mulch the bamboo forest with organic material in winter, which allows the farmer to harvest the shoots in the March earlier than expected (Mulumba and Lal, 2008). However, after 3-4 years of continuous mulch management, the *P. praecox* forest will inevitably experience an overall decline, resulting from degradation of the underground rhizome system, reductions in bamboo shoot production and quality, and flowering of the bamboo (Guo et al., 2011; Chen et al., 2014). According to prior studies, organic material mulching is the critical factor in the forest degradation since the fermentation of organic matter increased the temperature, blocked air exchange, and depleted the oxygen in the rhizosphere soil, resulting a low oxygen environment (Gui et al., 2013; Qian et al., 2020). Therefore, figuring out how to improve soil oxygen is the key to alleviating the bamboo degradation during the mulching period. Most of the methods currently used to alleviate root hypoxia are chemical methods that involve adding calcium peroxide (CaO_2_) or magnesium peroxide (MgO_2_), or quick release formulations like hydrogen peroxide (H_2_O_2_) or carbamide peroxide (CH_4_N_2_O·H_2_O_2_) (Liu et al., 2012; Liu and Porterfield, 2015), which largely changes the soil structure and the original living environment of the plants. Other methods require manually inserting tubes into the soil to increase soil oxygen levels, which is time-consuming and labor-intensive (Qian et al., 2022). Therefore, we devised a method using subsurface pipe aeration to investigate whether it could effectively increase oxygen levels in the root zone, improve plant physiological and biochemical performance, and thus mitigate degradation of the bamboo forest.

Hypoxic stress occurs frequently in nature (Drew, 1997; Bailey-Serres and Voesenek, 2008; Gibbs et al., 2011b). In order to survive, cells switch from aerobic respiration to anaerobic fermentation, generating harmful metabolites including lactic acid, acetaldehyde, and ethanol (Bailey-Serres and Voesenek, 2008c; Visser and Voesenek, 2006). Ethanol and lactic acid fermentation are the two primary metabolic routes for energy production under hypoxic conditions (Fukao and Bailey-Serres, 2004). The pyruvate content then increases, and the anaerobic enzymes alanine aminotransferase (AlaAT), pyruvate decarboxylase (PDC), alcohol dehydrogenase (ADH), and lactate dehydrogenase (LDH), are produced in these “hypoxic” cells (Armstrong et al., 2019). PDC and ADH catalyse the conversion of pyruvate PDC and ADH to ethanol, followed by the conversion of pyruvate to lactate by LDH and the simultaneous oxidation of NADH to NAD+ (Robertson et al., 1994; Kathleen et al., 2003). The reversible interconversion of alanine and 2-ketoglutarate to pyruvate and glutamate is catalyzed by AlaAT in the presence of higher pyruvate concentrations (Ricoult et al., 2006). Hu et al. (2005) found that ADH and PDC activities increased in roots of cucumber under anaerobic condition. In *Arabidopsis* and *Medicago truncatula*, LDH and AlaAT fermentation are enhanced in anoxic and hypoxic cells (Bray et al., 2002; Dolferus et al., 2008). Plant tolerance to hypoxia stress can be improved by increasing anaerobic enzyme activity (Gibbs et al., 2000; Morimoto and Yamasue, 2007). Therefore, anaerobic enzyme activity is also an important indicator.

Plant hormones including gibberellin (GA), abscisic acid (ABA), ethylene (ETH) and the growth hormone, indoleacetic acid (IAA), interact with each other to regulate biochemical and physiological processes (Davies, 2005; Achard et al., 2008; Bari and Jones, 2009; Santner et al., 2009; Peleg and Blumwald, 2011). Ethylene is the most sensitive hormone under hypoxic stress. Under anoxic conditions such as flooding, ethylene production is induced in the plant, which to some extent stimulates the formation of adventitious roots and air chambers, creating favorable conditions for nutrient and water uptake as well as oxygen input. Moreover, ethylene alleviates the toxic effects of secondary metabolites (Fukao et al., 2006; Hartman et al., 2021). The precursor of ETH, 1-aminocyclopropane-1-carboxylate (ACC), is formed by the action of ACC synthase (ACS) using methionine as the substrate, and ETH is then formed by the reactions catalyzed of ACC oxidase (ACO) with molecular oxygen as the coenzyme (Chae and Kieber, 2005; Rieu et al., 2005; Xu and Zhang, 2015). ACO and ACS are important rate-limiting steps in ETH synthesis (Barry et al., 1996; Vriezen et al., 1999). In addition, GA has been shown to stimulate seed germination and root elongation, while ABA is a potent inhibitor of GA activity (Holdsworth et al., 2008). ETH promotes elongation plant development and adventitious root production by managing the dynamic stability of ABA and GA concentration to expand the contact area of plants with air for more oxygen (Steffens and Sauter, 2006; Rzewuski and Sauter, 2008). Plant growth hormones known as auxins, such as IAA, increase root initiation and postpone plant senescence (Nguyen et al., 2018). Tognetti et al. (2012) found interaction between IAA and other hormonal signals during stress adaptation, which might be mediated by changes in plant growth and development. In addition, it was also found that the high IAA/ABA ratio is associated to the activity of rhizome buds in *P. praecox* (Hu et al., 1996). Jasim and Merhij (2013) suggest that there are improvements in IAA, GA3, and Zeatine while a reduction in ABA under mulch + fertilizers. These findings suggest that endogenous hormones are vital in the formation of bamboo roots. The key hormonal changes that lead to bamboo forest degradation after long-term mulching with organic materials and the relationship between them are poorly studied and deserve further investigation.

Auxin/indole-3-acetic acid (Aux/IAA) proteins are transcription factors (TFs) that control auxin-responsive gene expression during plant development (Remington et al., 2004; Szemenyei et al., 2008; Chandler, 2016). *Aux/IAA* family member genes are often homologous in the same plant; for example, *SlIAA2*, *SlIAA13*, *SlIAA15*, *SlIAA16*, and *SlIAA20* genes in tomato are functionally similar and are significantly expressed during root development (Wu et al., 2012). Other studies have also found specificity in *Aux/IAA* gene functions, such as the expression of *AetIAA3*, *AetIAA11*, and *AetIAA26* in *Aegilops tauschii*, which are tissue-specific genes that are expressed specifically in pistils, seeds, and roots, respectively (Qiao et al., 2014). The majority members of the Aux/IAA TF family have been associated with lateral root growth. For example, the *AtIAA14* gene controls lateral root development in Arabidopsis (Fukaki et al., 2010). In wheat, *TaIAA1* regulates the development of important organs such as roots and tillers, and also flowering and leaf patterns (Singla et al., 2006). In addition, there is also crosstalk between the *Aux/IAA* family and ethylene, and it thus regulates plant growth. According to Li et al. (2015), ETH modifies alkaline stress-mediated root development inhibition by increasing the expression of *Aux1* and auxin-related genes, which enhances auxin accumulation. So yet, only four members of the *Auxin* family, *Aux1*, *Aux2*, *Aux3*, and *Aux4*, have been discovered in *P. praecox*. Whether the *Aux/IAA* gene family has crosstalk with GA and/or ABA to regulate root growth is still unknown for the organic material mulching system used in *P. praecox*.

In this study, the effects of subsurface pipe aeration on soil oxygen and root physiology and biochemistry of a *P. praecox* forest under organic material mulching were investigated. The aims of the study were to examine the hypotheses: aeration improves the soil condition and associated physiological and biochemical properties of bamboo forests. The obtained evidences are expected to provide a new direction for the sustainable cultivation of bamboo forests.

## 2 Materials and methods

### 2.1 Site location

The research was performed at the Panmugang Modern Forestry Demonstration Base of Zhejiang Agriculture and Forestry University, Zhejiang Province, China (119°58′ E, 30°29′ N). This area has a subtropical monsoon climate with an average annual temperature of 17.8°C, an average relative humidity of 70.3%, an annual precipitation of 1,454 mm, a frost-free period of 234 d, and 1,765 hours of sunshine per year. The relevant weather data is in **Table S1**. The agricultural area has a hilly environment with hills that are typically less than 150 meters high. The soil is classified as a Ferralsol since it is largely originated from quaternary sandstone parent material. Natural precipitation and soil water storage are the primary sources of agricultural productivity (Xu et al., 2017).

### 2.2 Experimental design

The experimental *P. praecox* plot had a stand density of 15,000 plants per hectare, the average diameter at breast height of the bamboo culm was 3.89 cm, and the ratio of the number of bamboo culms in each year was year 1: year 2: year 3 = 1:1.89:0.58. The experimental area of the forest was split into twelve 50 m × 50 m plots, with the treatment arrangement being a full block with three replicates for each treatment. The treatments were (1) control; (2) mulching; (3) control + aeration (aeration) and (4) mulching + aeration (M+A). On December 17, 2020, the surface of the bamboo forest was mulched with organic material to increase the temperature and moisture content, and the hulled bran that had not decayed was removed on March 24, 2021. Once mulch has been removed, the shoot yield was recorded. The following was the mulching procedure: Initially, 4,500 kg·ha^−1^ of chicken manure was spread to the soil surface. The chicken dung was subsequently covered with rice straw (3,750 kg·ha^−1^). Finally, rice bran (412.5 t·ha^−1^) was sprinkled on top to provide 15 cm of thickness. We randomly selected three plots that had been mulched for many years as aerated plots. The aeration measures were as follows: the holes were drilled in a straight line parallel below the ground at a depth of 50 cm (the bamboo rhizomes are mainly present in the 20-30 cm layer) in each plot at a spacing of 60 cm, and plastic ventilation pipes with an external diameter of 21 mm and a wall thickness of 1 mm were then inserted and connected in sequence, with small holes of 0.2 mm diameter every 30 cm in the wall for ventilation. An air pump was connected to the main pipe in each plot and the air was delivered by a compressor (AS7.5Hi, Quanzhou Jinba, China). The plots with aeration were aerated for the whole day. Samples were collected in March, June, September, and December 2021. In each sample plot, the bamboo root was sampled from a depth of 20-30 cm and taken to the laboratory for analysis. At the end of the experiment, the root system was scanned with the Winrhizo root analysis system and calculated for root length density, surface area density and volume density and record the diameter at breast height of the bamboo. Root samples were cleaned with distilled water, instantly dried, frozen in liquid nitrogen, and kept at -80°C until tested.

### 2.3 Sample analysis

#### 2.3.1 Determination of soil oxygen content and temperature

Soil oxygen concentration and temperature were measured using a fiber-optic oxygen meter and a soil temperature probe (Firesting O_2_, Pyro Science, Germany), calibrated at two points using saturated air (21% oxygen) and saturated Na_2_SO_3_ solution (0% oxygen) before use. For the test, the measuring probe and the soil temperature measuring probe were mounted on the oxygen meter at the same time. After selecting the measuring point at the soil profile (25 cm), the two probes were slowly and accurately inserted into the soil, covered with soil, and the soil was then allowed to return to its original state after one week before the oxygen content and temperature measurements were taken. Three replicate measurements were taken for each sample plot. The oxygen meter recorded both soil oxygen concentration and soil temperature, with the probes buried in the same way.

#### 2.3.2 Root activity assay

The 2, 3, 5-triphenyltetrazolium chloride (TTC) redox technique was applied to assess root activity (Li et al., 2004). In the dark at 37°C, 0.5 g root pieces were pulverized with 5 mL PBS (pH 7.0) and 5 mL 0.4% TTC. To finish the incubation, 2 mL 1 M H_2_SO_4_ was supplied after 2 h. After wiping the roots with filter paper, they were homogenized in a mortar with 5 ml ethylacetate and fixed to 10 ml with ethylacetate. After that, a spectrophotometer was used to measure absorbance at 485 nm (UVmini-1280, Shimadzu, Japan), the root activity was expressed by TTC reduction (mg·g^−1^h^−1^).

#### 2.3.3 Enzyme activity assays

Fresh root samples were extracted with LDH, AlaAT, ADH and PDC using 9 ml of 0.1 M phosphate buffer (pH 7.0). After centrifuging the mixtures at 14,000 g for 15 minutes at 4°C, the supernatant was collected and the anaerobic enzyme activity was determined using the appropriate assay [LDH (A020-2); AlaAT (C009-2-1); ADH (A083-2-1); PDC (A141-1-1), Nanjing Jiancheng Bioengineering Institute, China] (Qian et al., 2020; Gao et al., 2022). The formula is as follows:


$$\begin{array}{c} \text{ADH activity}\\ \left(\text{U}/\text{g FW}\right) \end{array}=\frac{\Delta {\text{A}}_{\text{m}}-\Delta {\text{A}}_{\text{b}}}{6.22\times 0.5}\times \frac{{\text{V}}_{\text{t}}}{{\text{V}}_{\text{s}}}÷\text{T}\times 1000÷\text{FW}$$

ΔAm: A_2_-A_1_ (OD value of sample)

ΔAb: A_2_-A_1_ (OD value of blank)

V_t_: Total volume of reaction solution (1.5mL);

V_S_: Sample size (0.05mL);

T: Reaction time (10 minutes);

FW: sample fresh weight.


$$\begin{array}{c} \text{PDC activity}\\ \left(\text{U}/\text{g FW}\right) \end{array}=\frac{\Delta {\text{A}}_{\text{m}}-\Delta {\text{A}}_{\text{b}}}{\text{ϵ}\times \text{d}}\times \frac{{\text{V}}_{\text{t}}\times 10^{6}}{\text{W}\times {\text{V}}_{\text{s}}÷{\text{V}}_{\text{ts}}}÷\text{T}=1.61\times (\Delta {\text{A}}_{\text{m}}-\Delta {\text{A}}_{\text{b}})÷\text{w}$$

ΔAm: A_2_-A_1_ (OD value of sample)

ΔAb: A_2_-A_1_ (OD value of blank)

V_t_: Total volume of reaction system, 1 mL=0.001 L;


$$\text{LDH activity}\left(\text{U}/\text{g FW}\right)=\frac{{\text{A}}_{\text{m}}-{\text{A}}_{\text{c}}}{{\text{A}}_{\text{s}}-{\text{A}}_{\text{b}}}\times {\text{C}}_{\text{s}}÷\text{FW}$$

A_m_: Measured vials OD value;

A_c_: Control vials OD value;

A_s_: Standard vials OD value;

A_b_: Blank vials OD value.

C_s_: Standard solution concentration, 0.2 μmol/mL


$$\begin{array}{c} ALT\text{ activity of sample}\\ \left(\text{U}/\text{g FW}\right) \end{array}={\text{U}}_{\text{h}}÷\text{FW}$$

U_h_: The ALT activity of the protein homogenate to be tested is obtained through the standard curve;

FW: sample fresh weight.

#### 2.3.4 Hormone analysis

ELISA plant hormones assay kit were used to determine the concentrations of GA, IAA, ABA, ACO and ACS (Shanghai Enzyme-linked Biotechnology Co., Ltd., China). Horseradish peroxidase enzyme-catalyzed label-antibody complexes were formed by combining antibodies directed against GA, IAA, ABA, ACO and ACS with enzyme-catalyzed label and hormones, which generates a blue material when combined with TMB substrate solution. Spectrophotometric measurements were then performed at 450 nm (Infinite M200 pro, Tecan, Switzerland) (Gao et al., 2022; Li et al., 2022). In the Excel worksheet, the linear regression curve was plotted using the standard concentration as the horizontal coordinate and the corresponding OD value as the vertical coordinate, and the concentration value of each sample was calculated according to the curve equation.

Based on Gao et al. (2022), root samples of *P. praecox* were put in 15-mL glass vials with 1mL 0.6% water agar and closed instantly. Following a 4-hour dark incubation period at 30°C, 1 mL of gas was attracted from the air space of each vial with an air-tight syringe (Focus GC, Thermo, Massachusetts, USA) and infused into a gas chromatograph (Focus GC, Thermo) fitted with a capillary column (CP-CarboPLOT P7, California, USA) and flameion. The ETH production was then determined using the fresh weight (f.wt) of bamboo roots (Wu et al., 2011; Zhu et al., 2016).

#### 2.3.5 Quantitative real-time PCR (qRT–PCR) analysis

The OminiPlant RNA Kit was used to extract total RNA (CWBIO, CW2598, China). A spectrophotometer was applied to determine the concentration and purity of RNA (Nano Drop 2000c, Thermo Scientific, USA). To generate cDNA, the Prime ScriptTM RT reagent Kit with gDNA Eraser was utilized (Takara Bio, RR047A, Japan). Primers of *Actin*, *Aux1*, *Aux2*, *Aux3*, and *Aux4* came from Gao et al. (2022); primer of *PeNTB* was cited from Fan et al. (2013). In qRT-PCR assays, gene-specific primers of *Actin*, *Aux1*, *Aux2*, *Aux3*, and *Aux4* were utilized (**Table 1**). Ct values of *Actin* were used as internal controls. Values reported represent the averages of three biological replicates with two independent trials. Sangon Biotech produced the primers (Shanghai, China). The Ultra SYBR Mixture (Takara, RR820A) fluorescent dye was utilized for qRT-PCR (Applied Biosystems QuantStudio 6, USA). The 2^−ΔΔCT^ approach was then used to determine the relative gene expression levels (Livak and Schmittgen, 2001; Gao et al., 2022).

**Table 1:** Specific primers used for qRT-PCR.

Gene name	Primer sequence (5’-3’)	Amplicon size (bp)
*PeNTB*	F: TCTTGTTTGACACCGAAGAGGAG R: AATAGCTGTCCCTGGAGGAGTTT	133
*Actin*	F: CGTCAAAGCCCCAAGAACAC R: GCTAGGAAAGACAGCCCTGG	129
*Aux1*	F: GTTCGTGAAGGTGAGCATGG R: CGTTCATGCCGTTCATCCCT	155
*Aux2*	F: TCTGAGGATGTACGGAGGGT R: GCATCAGATCGCCGTCCTTG	125
*Aux3*	F: AAGGGCATGAACGAGAGCAA R: CGACTCGACGAACATCTCCC	126
*Aux4*	F: TGACCAGCCGATGACGAAG R: GCTGCTTGGAAGGTGTTCCT	186

### 2.4 Statistical analysis

Using SPSS 20.0, all data were statistically assessed utilizing ANOVA and Duncan’s Multiple Range test (IBM Corp., Armonk, NY, USA). Correlation and redundancy analyses were carried out using R program v3.6.3. Origin v8.0 was used to create the figures (Origin Lab Corporation, Northampton, USA).

#### 2.4.1 Redundancy analysis (RDA)

RDA is a method that combination of correspondence analysis and multiple regression analysis, each step of the calculation is regression with environmental factors, also known as multiple direct gradient analysis (Borcard et al., 1992; Larkin et al., 2007). This analysis is used to reflect the relationship between genetic (*Auxs/IAA*) and enzyme and hormones factors in this study. Results were visualized by RDA biplot using CANOCO (version 4.5), where the position, angle, and length of arrows indicated the direction, degree, and scope of response of the genetic (enzyme and hormones) to enzyme and hormones (genetic) variables. The main function of the Monte Carlo test (Julian and Peter, 1989) is to test the significance of the constrained ranking method.

#### 2.4.1 Correlation analyses

Pearson correlation analyses between enzyme and hormones and gene characteristics were performed using SPSS Statistics v20.0 (IBM Corp., USA) and illustrated using the “ pheatmap” package in R v 4.0.2. Before variance analysis, we used Shapiro-Wilk and Levene tests to assay the data normality and the equality of variances, respectively. We conducted a one-way Analysis of Variance to explore the effects of aeration on plant enzyme and hormones and gene characteristics using SPSS Statistics v20.0. F values were derived from ANOVA at *p< 0.05, p< 0.01*, and *p< 0.001* using SPSS v20.0.

## 3 Results

### 3.1 Effect of aeration on bamboo growth under coditions

**Figure 1:**
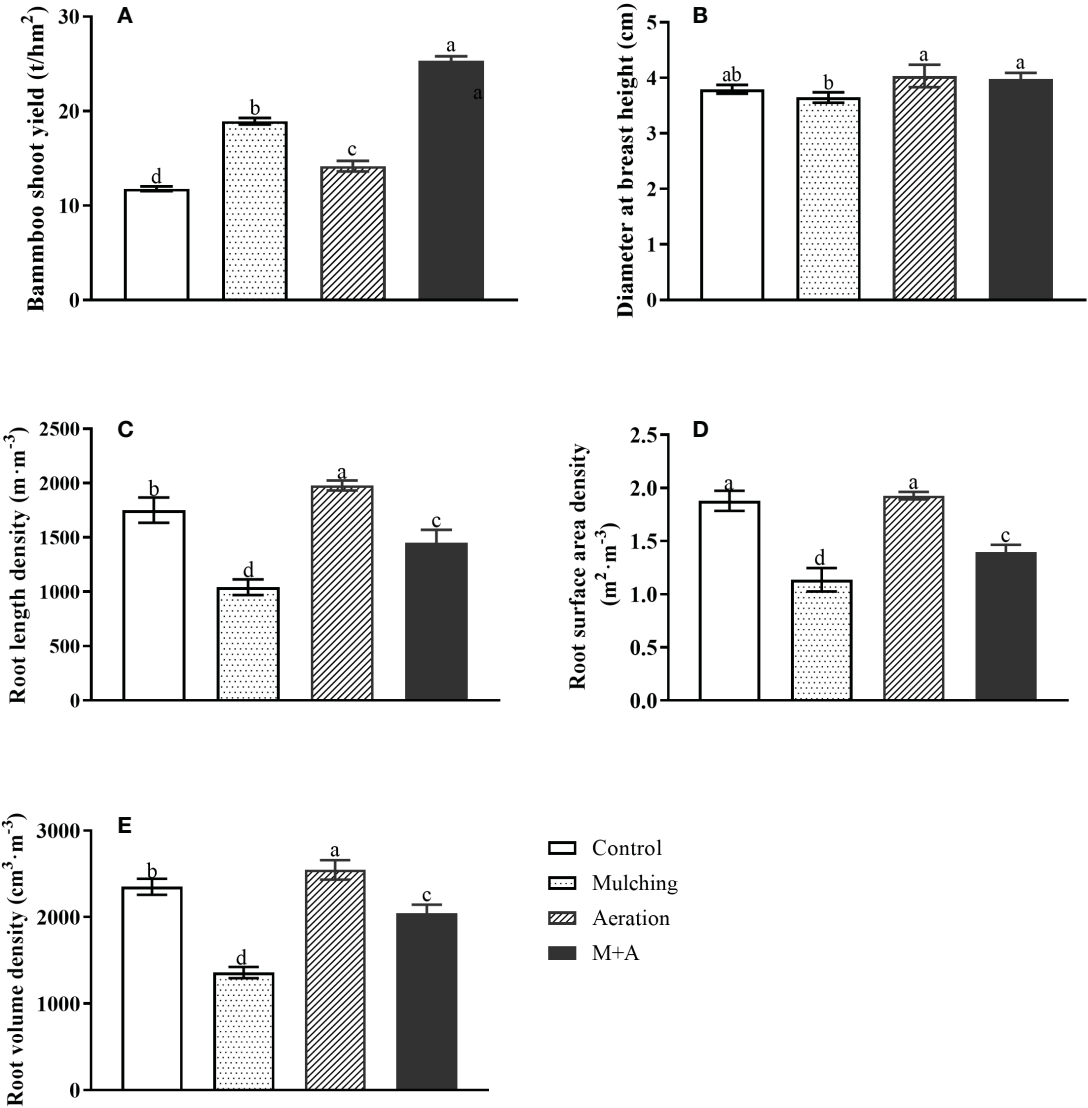
Effects of aeration on shoot production **(A)**, diameter at breast height **(B)**, root length density **(C)**, surface area density **(D)** and root volume density **(E)** in the bamboo forest under mulching conditions (Control, Mulching, Aeration, M + A). The vertical bars ± reflect the standard deviation of the mean. Different letters in the same period represent different significance (*p<* 0.05).

Compared to the control, shoot yield was significantly increased by 60.5%, 20.23% and 115.1% for mulching, aeration and M+A respectively, and by 34.0% for M+A compared to mulched (**Figure 1A**). As for diameter at breast height, the diameter at breast height in the aeration group was significantly increased compared to the mulched group (**Figure 1B**). Mulching significantly reduced root length density (67.9%), root surface area density (39.4%), and root volume density (73.0%), respectively, compared to the control (**Figures 1C–E**). But M+A significantly increased root length density (39.3%), root surface area density (22.7%), and root volume density (50.6%), respectively, compared to mulching. It showed that mulching combined with aeration techniques has a positive effect on the growth of bamboo.

### 3.2 Effect of aeration on soil oxygen concentration, soil temperature, and root activity under mulching conditions

**Figure 2:**
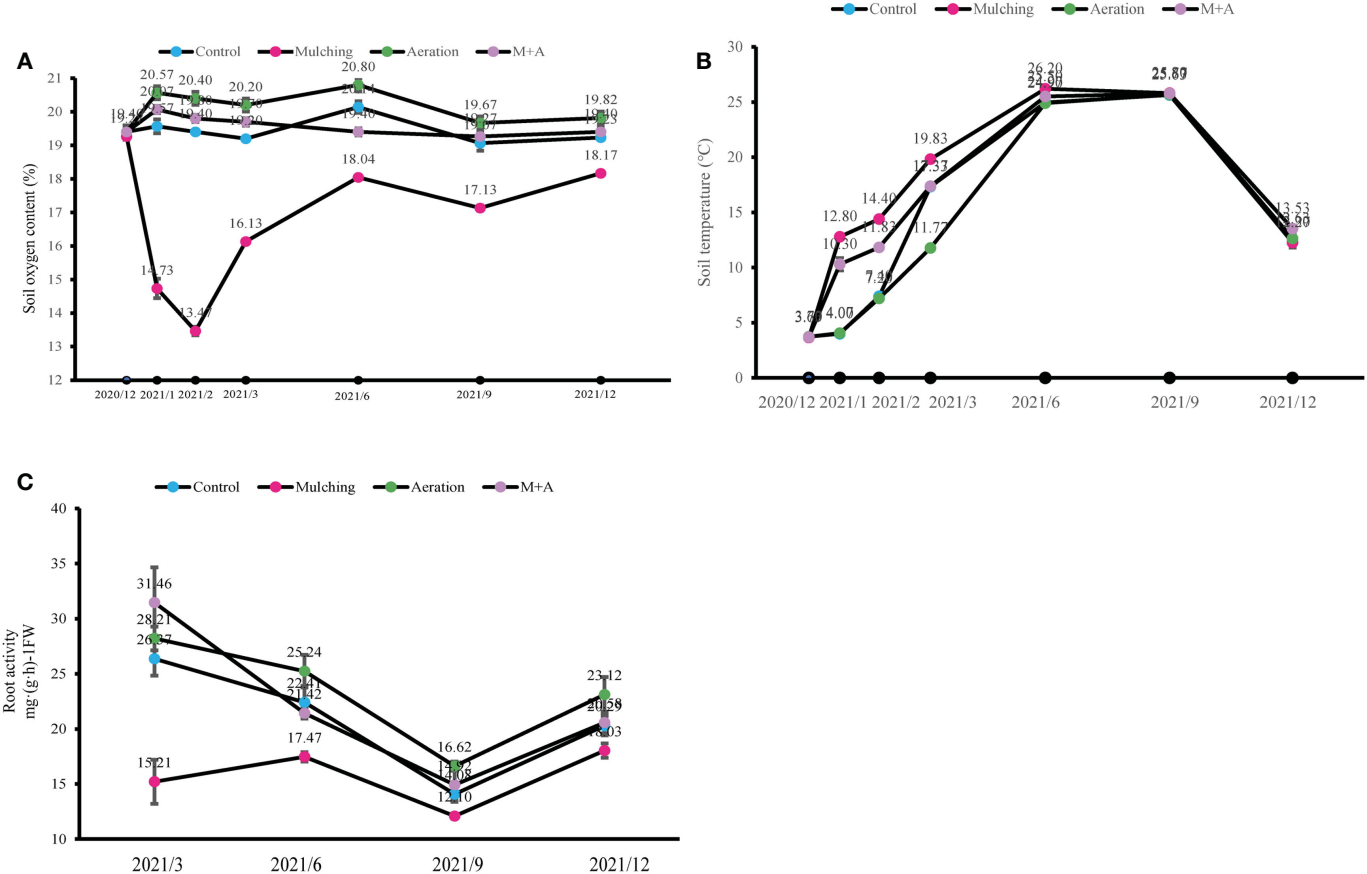
Effects of aeration on soil oxygen content **(A)**, temperature **(B)** and root respiratory activity **(C)** in the bamboo forest under mulching conditions (Control, Mulching, Aeration, M + A). The vertical bars ± reflect the standard deviation of the mean. Different letters in the same period represent different significance (*p<* 0.05).

When the bamboo mulched, soil oxygen concentration decreased rapidly, reaching its lowest level after two months (**Figure 2A**). Three months after mulching, soil oxygen concentration began to recover when the mulch was removed, but it was still lower than that of the control plots. There were no significant differences in oxygen concentration between the treatments of M+A and control in June, September and December. During the mulching period, soil temperature in the mulched treatments was significantly higher than the control, while aeration significantly reduced the soil temperature compared to the mulched (**Figure 2B**). When the mulch was removed in March, soil temperature increased and then decreased with time that was consistent with the air temperature. From June, there were no significant differences among all treatments. As indicated by **Figure 2C**, the mulching resulted in a lower root activity compared to the control, while aeration improved the activity significantly during the mulching peroid.

### 3.3 Effect of aeration on anaerobic enzyme activity in roots under mulching conditions

**Figure 3:**
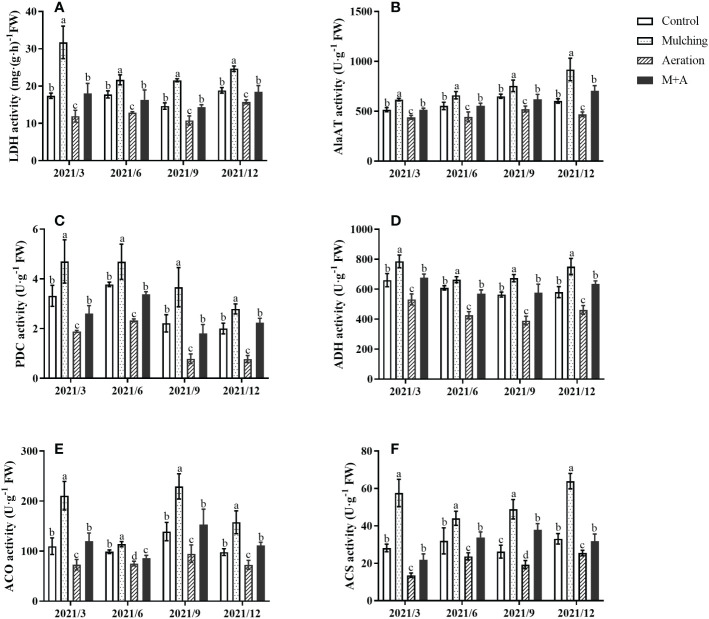
Effects of aeration on the activities of LDH **(A)**, AlaAT **(B)**, PDC **(C)**, ADH **(D)** ACO **(E)** and ACS **(F)** in the bamboo forest under mulching conditions (Control, Mulching, Aeration, M + A). The vertical bars ± reflect the standard deviation of the mean. Different letters in the same period represent different significance (*p<* 0.05). LDH, lactate dehydrogenase; AlaAT, alanine transaminase; PDC, pyruvate decarboxylase; ADH, alcohol dehydrogenase; ACO (ACC oxidase) and ACS (ACC synthase).

LDH, AlaAT, PDC, and ADH activities of mulching treatment were significantly elevated and the M+A treatment significantly lowered the activities of these anaerobic enzymes compared with mulching throughout the year (**Figures 3A–D**). There was no significant difference between the M+A and the control regarding anaerobic enzyme activity. Anaerobic enzyme activity of aeration group was significantly decreased compared to the control.

### 3.4 Effect of aeration on the activities of ACO and ACS in roots under mulching conditions

Mulching significantly improved the activities of ACO and ACS, aeration significantly reduced both activities (**Figures 3E, F**). For all four seasons, there were no statistically significant changes between the aeration treatment and the control. The activities of ACS and ACO were lower overall in June when compared to the other months of the year.

### 3.5 Effects of aeration on the hormone content in roots under mulching conditions

**Figure 4:**
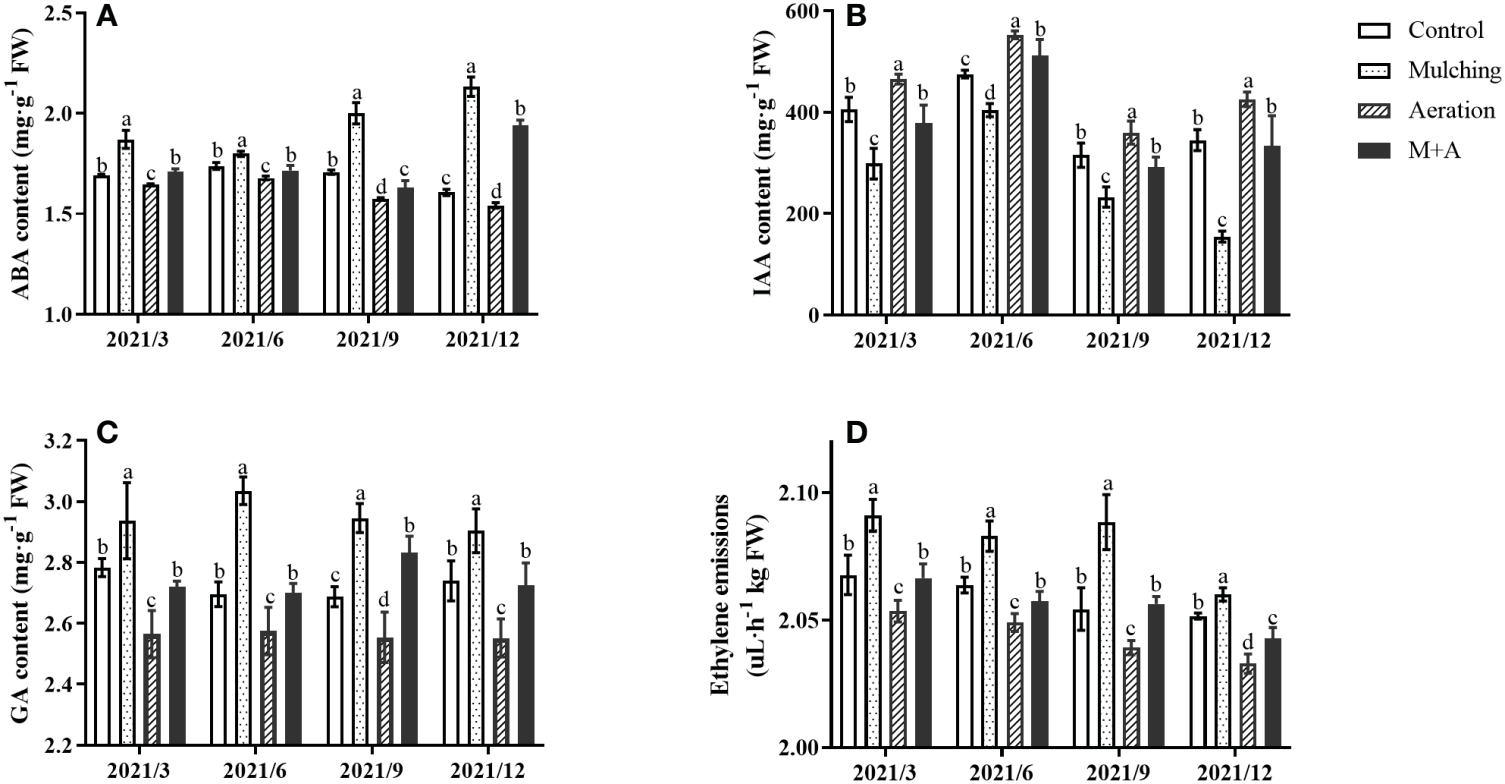
Effects of aeration on the contents of the phytohormones ABA **(A)**, IAA **(B)**, GA **(C)**, and ETH **(D)** in the bamboo forest under mulching conditions (Control, Mulching, Aeration, M + A). The vertical bars ± reflect the standard deviation of the mean. Different letters in the same period represent different significance (*p<* 0.05). ETH, ethylene; ABA, abscisic acid; GA, gibberellic acid; IAA, indole-3-acetic acid.

In March and September, mulching significantly increased ABA, GA, and ETH contents in the roots and decreased the IAA content compared to the control, while aeration significantly decreased the contents of ABA, GA, and ETH and increased the IAA content in the M+A group (**Figures 4A–D**). ABA, GA, and ETH contents were significantly reduced and IAA was enhanced in aeration treatment compared to the control.

### 3.6 Effect of aeration on *Aux/IAAs* gene expression in roots under mulching conditions

**Figure 5:**
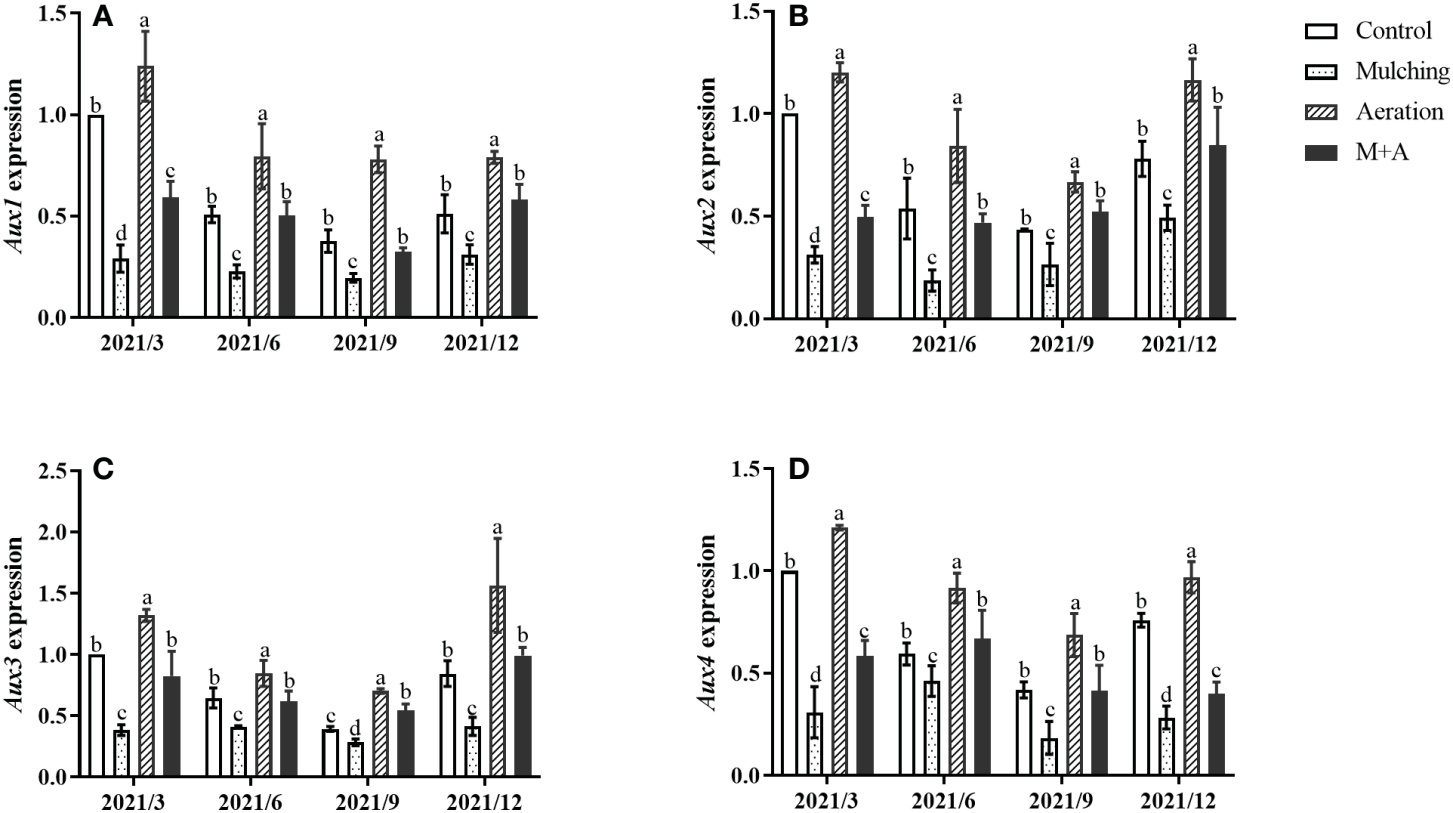
Effects of aeration on the expression of the *Aux1* **(A)**, *Aux2* **(B)**, *Aux3* **(C)** and *Aux4* **(D)** genes in the bamboo forest under mulching conditions (Control, Mulching, Aeration, M + A). The expression levels of *PeNTB* and *Actin* were used to normalize the expression for each sample. The vertical bars ± reflect the standard deviation of the mean. Different letters in the same period represent different significance (*p<* 0.05).

Mulching significantly reduced the expression of *Aux1*, *2, 3*, and *4* compared to the control throughout the year, while soil aeration significantly enhanced *Aux* gene expression in the M+A group (**Figures 5A–D**). *Aux/IAAs* gene expression of aeration was significantly increased compared to the control.

### 3.7 Analyses of correlations between enzyme and hormones and gene expression traits

**Figure 6:**
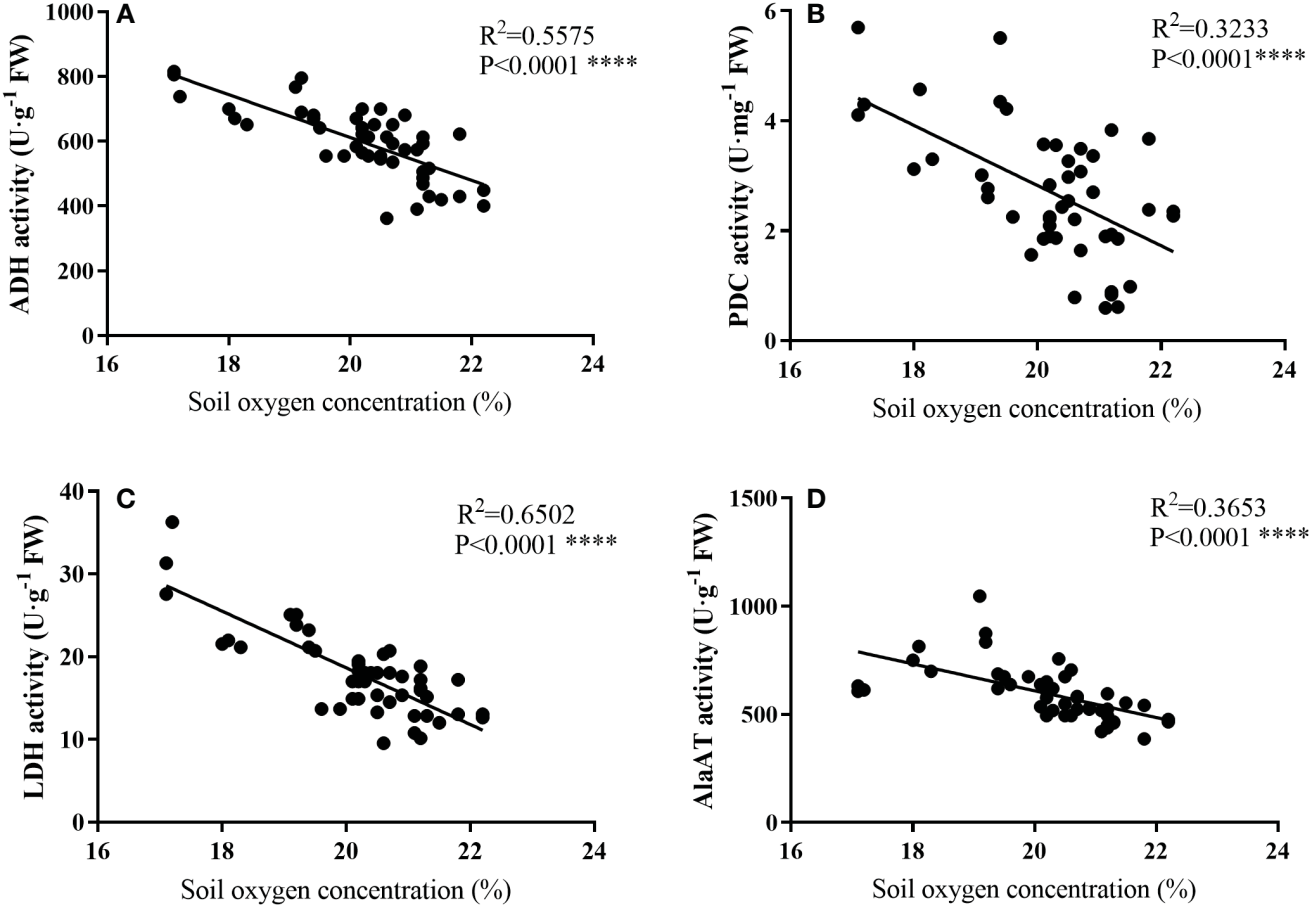
Relationships between soil oxygen concerntration (%) and the activities of ADH **(A)**, PDC **(D)**, LDH **(C)**, and AlaAT **(D)** of bamboo roots under mulching conditions (Control, Mulching, Aeration, M + A). Data are from Figures 1 and 3 and represent mean values. Regression equations are Y = -66.22X_1_ + 1936, Y = -0.5478X_2_ + 13.78, Y = -3.426X_3_+ 87.18 and Y = -62.03X_4_ + 1850, respectively, for **(A–D)** (Y is the soil oxygen concerntration, and X_1_, X_2_, X_3_, and X_4_ represent ADH, PDC, LDH, and AlaAT, respectively). R^2^ is determined as the coefficient of determination. (*p*< 0.01, n=48).

As shown in **Figure 6**, the activities of ADH, PDC, LDH, and AlaAT all showed a linear correlation with soil oxygen concentration, and the activities of anaerobic enzyme increased as the oxygen concentration decreased.

**Figure 7:**
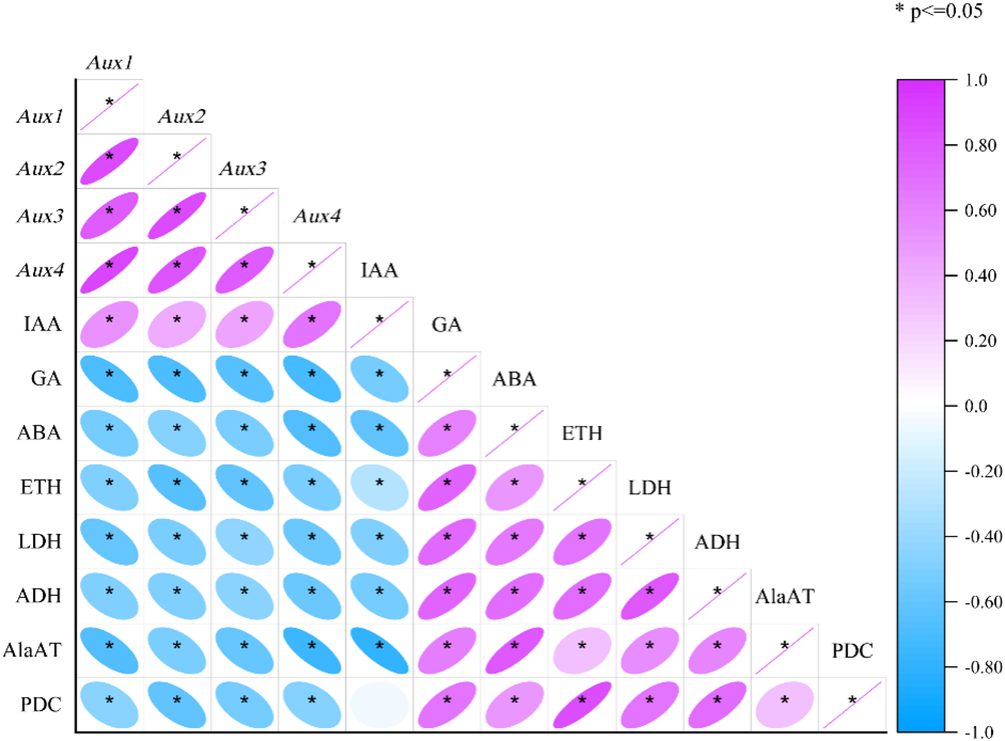
Correlation matrix of physiological and gene factors of the roots cultivated with and without mulching and aeration. PDC: pyruvate decarboxylase; AlaAT: alanine aminotransferase; LDH: lactate dehydrogenase; ADH: alcohol dehydrogenase; IAA: indole-3-acetic acid; ETH: ethylene; ABA: abscisic acid; GA: gibberellic acid and *Aux1*, *2*, *3*, *4*: auxin-related genes 1, 2, 3 and 4, respectively.

Pearson’s correlation tests were also carried out to assess the correlations between each enzyme and hormones and genetic attribute under mulching and aeration (**Figure 7**). The expression of *Aux1*, *2*, *3*, and *4* was substantially positively associated with IAA levels, and *Aux/IAAs* expression was significantly negatively associated with ETH, ABA and GA concentration (*p<* 0.05). Moreover, ETH was significantly positively related with the activities of PDC, ADH, LDH, AlaAT, and also with ABA and GA concentration. ABA concentration was significantly positively correlated with anaerobic enzyme activities and ETH and GA concentration, and significantly negatively correlated with *Aux/IAAs* expression and IAA concentration. As for anaerobic enzyme activity, LDH activity was significantly positively associated with the activities of PDC, AlaAT, and ADH, and the concentrations of ABA, ETH, and GA and significantly negatively correlated with IAA concentration and *Aux/IAAs* gene expression (*p<* 0.05). ADH, LDH and AlaAT activity was significantly positively associated with anaerobic respiration enzymes activities and ETH, GA, and ABA concentrations, and significantly negatively associated with IAA and *Aux/IAAs* gene expression (*p<* 0.05). The activity of PDC was significantly positively correlated with ETH, ABA and GA concentrations and the activities of anaerobic respiration enzymes, and it was significantly and negatively associated with *Aux/IAAs* gene expression (*p<* 0.05). The analysis showed that plant hormones have an important role in mulch-induced root hypoxia in *P. praecox*, influencing changes in *Aux/IAAs* expression and anaerobic enzymes activities.

### 3.8 Redundancy analysis of enzyme and hormones and gene parameters

**Figure 8:**
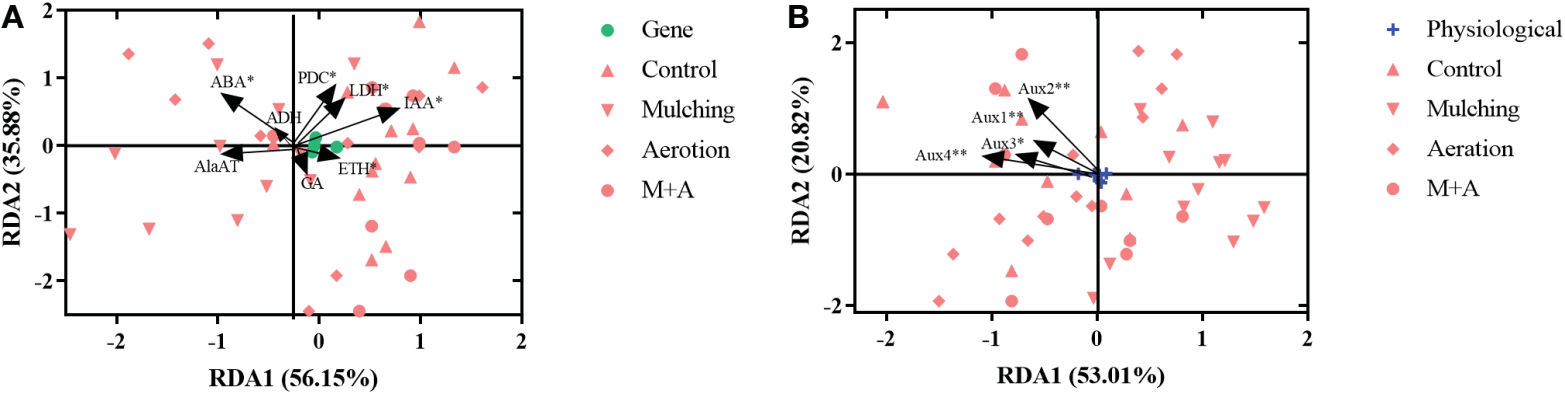
Redundancy analysis results reveal the correlations between physiological **(A)** and gene **(B)** factors in roots under mulching and aeration conditions. PDC: pyruvate decarboxylase; AlaAT: alanine aminotransferase; LDH: lactate dehydrogenase; ADH: alcohol dehydrogenase; IAA: indole-3-acetic acid; ETH: ethylene; ABA: abscisic acid; GA: gibberellic acid and *Aux1*, *2*, *3*, *4*: auxin-related genes 1, 2, 3 and 4, respectively.

We did redundancy analysis (RDA) to see whether there were any commonalities among the treatments in terms of enzyme and hormones (ADH, AlaAT, LDH, and PDC activities, hormone concentrations of ABA, IAA, GA, and ETH) and genetic characteristics (*Aux* gene expression) (**Figure 8**). As a result, we found that enzyme and hormones and genetic characteristics interact with one another. The activities of ABA, PDC, and LDH, as well as the concentrations of ETH and IAA, had a significant influence on plant genetic composition (*p<* 0.05), with RDA1 and RDA2 exhibiting variances across all treatments, accounting for 56.15 and 35.88% of the variation, respectively (**Figure 8A**). Furthermore, *Aux1*, *Aux2*, *Aux3*, and *Aux4* expression was strongly associated to plant enzyme and hormones parameters, with the first and second major axis accounting for 53.01 and 20.82% of the variance, respectively (**Figure 8B**).

## 4 Discussion

### 4.1 Aeration improved soil condition and bamboo growth under mulching

In natural environments, soil hypoxia is caused by factors such as heavy rainfall, poor soil structure, and little drainage, which generate unfavorable porosity and ventilation interactions in the soil, restricting root growth and crop output considerably (Brookes et al., 1982; Weits et al., 2019; Strudley et al., 2008). Nevertheless, when bamboo forest grove is mulched, the organic material heats and ferments. The aerobic microorganisms consume oxygen directly from the soil, and the thick mulching material stops ambient oxygen from entering the soil, which causes a lack of oxygen at the root level, unlike flooding and soil slumping (Jiang et al., 2009; Qian et al., 2020). We discovered in the investigation that soil temperatures increased and soil oxygen concerntration decreased during the mulching period (**Figure 2A, B**; **Table 2**), which is in line with previous research findings (Qian et al., 2020). However, aeration significantly reduced soil temperature, increased oxygen content, diameter at breast height, shoot production and root growth, suggesting that soil aeration using buried pipes is effective in improving soil condition and bamboo growth. After removal of the mulch, soil oxygen concentrations recovered substantially but remained slightly lower than in the control. Also, soil temperatures did not differ significantly between the three treatments from June onwards. It is possible that mulching changed the soil microbial populations and soil structure, but after the organic material was removed, the soil repaired itself and gradually returned to the control level. Root growth is known to be limited by low soil oxygen availability (Christianson et al., 2010; Cruz et al., 2019). Hence, we found that mulching reduced root activity while aeration increased it. Also, it has stronger root activity in March than the other months (**Figure 2C**; **Table 2**). It is similar to previous research findings, Holthausen and Caldwell (1980) suggested that root system’s breathing capacity varies seasonally, meaning that respiratory capacity peaks in spring and a respiratory minimum occurs in late summer. This tendency might be attributed to environmental pretreatment together with an overall genetic-based program to extend the length of root activity and reduce the root system’s carbon requirement (Holthausen and Caldwell, 1980).

**Table 2:** The F value is obtained from the analysis of variance (ANOVA) on the data of the factors in the roots when different aeration measures were applied under mulching. *, **, *** significant at 0.05, 0.01 and 0.001 probability, respectively.

Source of variation	df	Soil O_2_ content	Soil temperature	Root activity	PDC	AlaAT	ADH	LDH	ETH	ABA	GA	IAA	*Aux1*	*Aux2*	*Aux3*	*Aux4*
Time-M	3	22.87***	7.05***	11.82***	0.81	4.86*	3.07	4.25*	7.31*	20.21***	0.26	7.31**	6.50**	5.09*	9.04***	7.32**
Mulching	1	428.03***	30.20***	86.20***	30.2***	59.03***	61.21***	136.33***	63.61***	125.17***	49.66***	134.01***	105.60***	60.97***	109.34***	184.88***
Time-A	3	0.95*	0.37	0.14	3.03*	0.73	1.10*	3.95*	0.23	9.17***	0.12	1.22*	1.42*	4.12*	5.81**	6.67**
Aeration	1	69.55***	0.01	27.92***	226.09***	82.17***	119.04***	122.14***	60.57***	180.26***	35.73***	64.62***	51.69***	71.41***	66.79***	117.67***
Time-M+A	3	25.81***	10.35***	6.38**	2.35*	0.73	0.14	4.77**	1.96*	9.62***	0.80	6.98**	1.36*	1.93*	3.09**	1.84*
M+A	1	423.01***	2.05*	70.77***	36.36***	55.56***	51.30***	89.80***	114.40***	126.28***	39.12***	54.90***	97.97***	68.27***	93.18***	31.53***
Mulching × time	3	17.58***	1.05*	11.86***	1.05*	5.131*	2.78	11.09***	4.75*	18.74***	0.37	5.89**	9.40***	3.07	10.93***	8.55***
Aeration × time	3	0.81	0.46	0.49	0.47	0.97*	0.44	1.63*	0.22	9.26***	0.11	0.14	0.21	3.14	6.78**	1.33*
M+A × time	3	26.84***	9.83***	11.74***	1.97*	3.09**	0.33	4.68**	1.99*	9.77***	0.70	2.40**	24.60***	1.92***	4.46**	0.822*

### 4.2 Aeration changed anaerobic enzyme activity under mulching

Pyruvate produced by glycolysis will undergo anaerobic respiration (also called fermentation) in plant cells once the oxygen content is low (Yazdani and Gonzalez, 2007). The reversible conversion of alanine and 2-ketoglutarate to pyruvate and glutamate is catalyzed by AlaAT (Ricoult et al., 2005). Fermentation may be classified into two types: lactic acid fermentation, in which LDH is responsible for catalyzing the transformation of lactate to pyruvate then back, with the end product being lactate; and alcoholic fermentation, in which ADH catalyzes the acetaldehyde-to-ethanol conversion. PDC catalyzes the oxidative decarboxylation of pyruvate to acetyl-CoA and NADH, with carbon dioxide and ethanol as byproducts (Bailey-Serres and Chang, 2005; Arbona et al., 2010; Armstrong et al., 2019). Neither type of fermentation produces ATP molecules and both are detrimental to cell survival (Bailey-Serres and Chang, 2005). Our results showed that the activities of PDC, LDH, AlaAT and ADH were significantly increased of bamboo root during the mulching period (**Figure 3A-D**; **Table 2**). This discovery is in line with the findings of Qian et al. (2020). After the mulch was removed, anaerobic enzyme activity of mulching remained greater than in the control, but aeration drastically decreased anaerobic enzyme activity. It occurred because the mulching technique lowered soil oxygen concentration and enhanced anaerobic enzyme activity. There was a linear relationship between the activities of the anaerobic enzymes ADH, PDC, LDG, and AlaAT and soil oxygen concerntration (**Figure 6**; **Table 2**). Extensive molecular and biochemical analyses revealed the mechanism behind these relationships. In hypoxic tissues in barley (*Hordeum vulgare*) and *M. truncatula*, AlaAT activity and gene expression are stimulated (Muench and Good, 1994; Bray et al., 2002; Ricoult et al., 2005). Previous studies have shown that flood-tolerant plants accumulate alanine by activating AlaAT, and that the alanine is carried *via* the xylem and becomes a transportable energy source (De Sousa and Sodek, 2003). AlaAT is essential for plant life not only in hypoxia, but also throughout the reoxygenation period following hypoxia (Nakamura and Noguchi, 2020). Additionally, when the oxygen content was inadequate in the root zone, the activities of LDH, PDC, and ADH, as well as the expression of the genes that encode these enzymes, were elevated in cucumber (Xu et al., 2014). Increased anaerobic enzyme activity may enhance plant tolerance to hypoxia (Kato-Noguchi, 2001). For example, plants of white clover with strong ADH activity, demonstrate better flood tolerance under flood stress than plants with weak ADH activity (Chan and Burton, 1992). Generally, subsurface buried pipe aeration reduced the anaerobic enzyme activity of bamboo roots caused by mulching with organic materials.

### 4.3 Aeration regulated hormone variation under mulching

The levels of some phytohormones in *P. praecox* are highly susceptible to external environmental conditions, and the insulating effect of mulching disrupts the balance of endogenous hormones. Endogenous plant hormones, including IAA, GA, ABA, and ETH, are the “switches” that modulate and control plant growth (Davies, 2004). A previous study showed that lateral shoots at the base of bamboo plants had significantly higher IAA/ABA and ZT/ABA levels one year after mulching than did plants grown without mulching, thus promoting early differentiation of lateral shoots (Huang et al., 2002). However, this does not correspond with our experimental results, where we found significant increases in GA and ABA contents and a reduction in growth hormone content of roots treated with organic material mulch for consecutive years, and this was also found in degraded *P. praecox* stands (**Figure 4**; **Table 2**). The ABA, GA, and cytokinin (CTK) contents of flowering bamboo in the degraded *P. praecox* forest were all higher than in unflowered bamboo, with the most significant increase being in ABA content (He et al., 2005). It might be because long-term mulching inhibited plant root growth, but the anoxic environment caused by mulching allowed the roots of bamboo to stretch towards soil surface to find more oxygen, thus increasing the contents of ABA, which promotes the formation of plant organ separation, and GA, which promotes cell elongation and division, ultimately leading to degradation of the bamboo forest (Davies, 2004). Previously, Xu et al. (2017) also found that long-term mulching caused roots to grow toward the ground in search of oxygen, which promoted root elongation. This phenomenon is also observed in rice. Deep-water rice leaves and internodes may stretch and grow above the water surface under flood circumstances to gather oxygen and prevent drowning (Ayano et al., 2015). We also found that the higher ABA contents in September and December and the higher IAA contents in March and June may be related to the growth habit of the plant (**Figure 4A, B**; **Table 2**), where the plant grows vigorously in spring and summer, while abscisic acid inhibits germination and promotes dormancy and plant organ separation in autumn and winter (Baktir et al., 2004; Davies, 2004). In addition, the actions of ABA and IAA are antagonistic, and one study showed that ABA may function as an inhibitor of GA and restrict root development, allowing the plant to survive during flooding (Wu and Hong, 2021). Our results suggest that there may be some antagonistic effects between ABA and IAA and GA in the hypoxic environment caused by organic material mulching (**Figure 7**; **Table 2**). In addition to this, it has been shown that the fast buildup of ethylene in submerged tissues (through physical trapping and active synthesis) under anoxic circumstances, causes alterations in branch lengthening, glucose metabolism, and adventitious root development (Steffens and Sauter, 2006; Xu et al., 2006; Hattori et al., 2009). At the same time, the equilibrium of GA and ABA contents is likewise coordinated by ETH under anoxic conditions caused by submergence (Xu et al., 2006). In this investigation, we discovered significant increases in ETH content and the activities of enzymes involved in ethylene synthesis (ACO, ACS), and also a significant positive correlation between ETH and GA contents under organic material cover (**Figures 3E, F**; **4D**; **7**; **Table 2**). It is due to the synergy established by the combination of ETH and GA. From this we can infer that ETH perception is essential for adventitious root development, and GA substantially promotes the ensuing ETH-induced adventitious root growth (Steffens and Sauter, 2006). Aeration from the buried pipes provided oxygen to alleviate soil hypoxia caused by mulching, thus changing the hormone contents in the bamboo roots by reducing the ABA, GA, and ETH contents, decreasing the activities of ACS and ACO, increasing the IAA content, and finally improving metabolic and physiological alterations in roots.

### 4.4 Aeration regulated *Aux/IAAs* gene expression under mulching

It is well known that auxin is the key regulator during plant growth (Wu et al., 2012; Eysholdt-Derzso and Sauter, 2017). IAA, a most abundant hormone in higher plants, is a weak acid, and growth hormone influx and efflux carriers promote its intercellular movement (Wu et al., 2012; Van and Licausi, 2015) Auxin transporters are necessary for the transfer of auxin into various cells. AUXIN1 (encoded by *Aux1*) is an auxin influx carrier. AUXIN1 is the major transporter for auxin uptake in root hairs and it controls root gravitropism, root hair formation, and leaf phyllotaxy (Ori, 2019). In this study, we found that mulching reduced the expression of *Aux1* (**Figure 5A**), and that *Aux1* expression was highly associated with growth hormone and unfavorably related to ETH concentrations (Li et al., 2015). *Aux2* and *Aux3* have been implicated in processes like as hypocotyl elongation and foliar growth in Arabidopsis and rice. *Aux3* regulates lateral root growth and root hair production, whereas *Aux4* regulates plant tiller height (Liscum and Reed, 2002; Overvoorde, 2005; Song and Xu, 2013). In the present study, mulching reduced gene expression of *Aux* genes, while aeration increased *Aux* gene expression (**Figure 5**). *Aux/IAAs* expression was favorably linked with IAA and negatively associated with ethylene (**Figures 7**, **8**). It is because ETH can control IAA synthesis by regulating *Aux1* expression and growth hormone synthesis-related genes, which in turn regulate root development under adverse situations (Li et al., 2015).

Following aeration, the correlations between each enzyme and hormones indicator and gene expression were also investigated. *Aux/IAAs* gene expression was highly related to many enzyme and hormones factors, and the expression of *Aux1*, *2*, *3*, and *4* was closely associated with root development factors (**Figure 8**). The results imply that *Aux/IAAs* genes are engaged in the management of hormone levels as well as the regulation of anaerobic enzymes and root respiration activity to keep proper root development, while *Aux/IAAs* contributing in this mechanism (Gao et al., 2022). Abiko et al. (2012) showed the expression of *Aux/IAA* in *Zea nicaraguensis* of hypoxic circumstances altered dramatically, and it may also govern the development of adventitious roots and the production of vented tissue. Mulching caused fast alterations in a number of critical enzyme and hormones markers in *P. praecox*. Here, LDH, PDC, ABA, IAA, and ETH all had significant impacts on the expression of *Aux/IAAs* (**Figure 8**). It demonstrates that the overlay affects *Aux/IAAs* expression in plants, which in turn regulates changes in endogenous hormone levels that are involved in regulating anaerobic respiratory enzymes and ultimately improving ability of plant roots to cope with hypoxia caused by organic materials.

**Figure 9:**
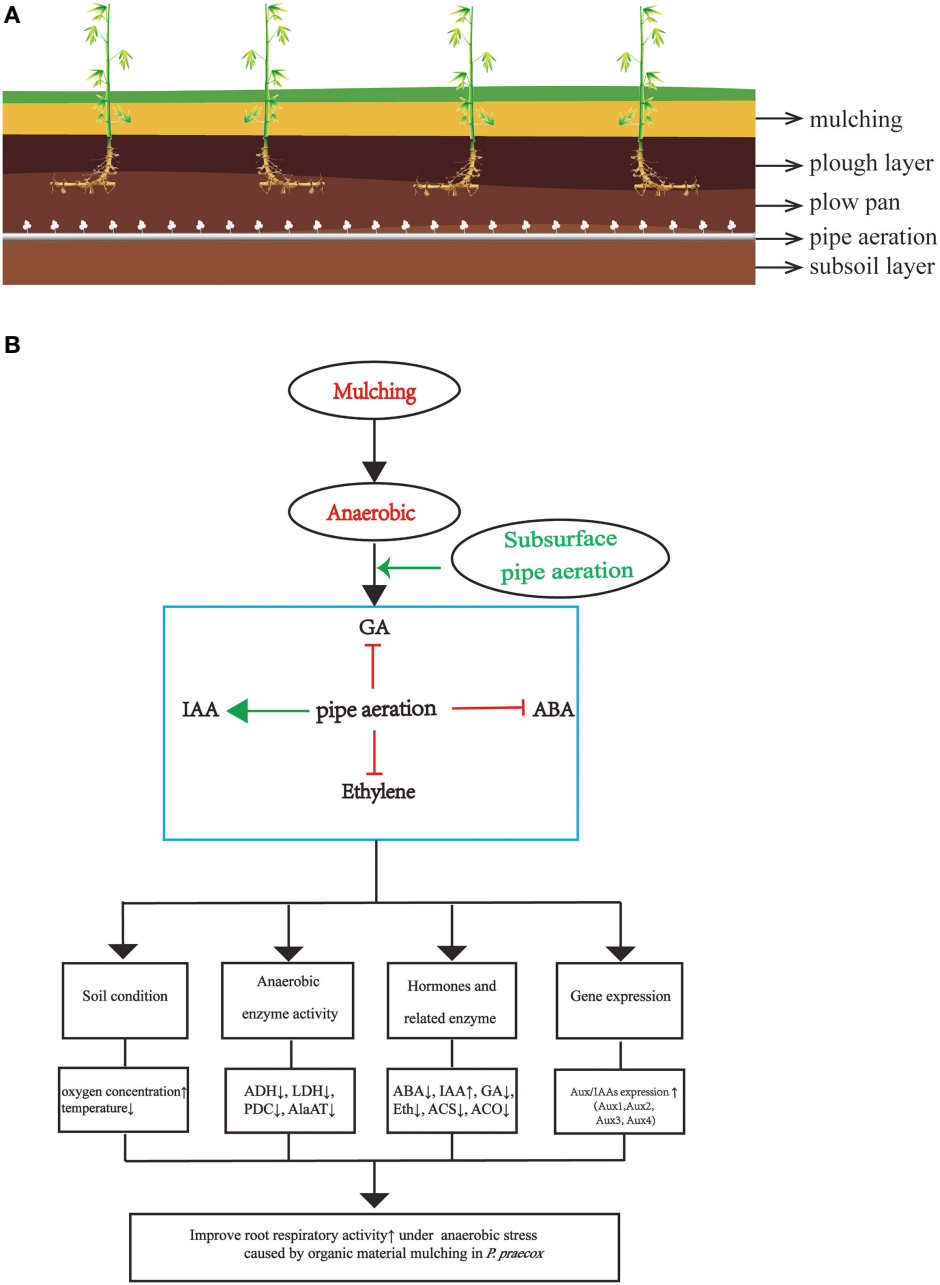
**(A)** the scheme of soil horizons including plants and air-pipe system; **(B)** a model for root aeration through hormone in *P. praecox*. Aeration promotes downstream protective systems in mulching *via* interacting with hormones (decreasing ABA, GA and ethylene content as well as increaseing IAA content), for instance, lowering soil temperature, anaerobic enzyme activity, increasing soil oxygen concentration, changing hormone synthase related activity, as well as increasing the expression of *Aux/IAAs* to stimulate root growth. Green arrow represents promotion, whereas red t-bars denote inhibition.

Overall, the findings of our investigation demonstrate that mulching with organic material degrades *P. praecox* forests (**Figure 9**), which consistent with the phenomenon observed informally by local farmers (Xu et al., 2017). We are here for the first time to demonstrate the mechanism of underground pipeline aeration to mitigate the degradation of bamboo forest. We also explain for the first time that hormones crosstalk with *Aux/IAAs* and thus regulate changes in enzyme and hormones indicators under bamboo forest mulching. We therefore are of the opinion that our subsurface aeration strategy will help to mitigate soil hypoxia and, in turn, improve the growth of bamboo. However, the intensity and time of the aeration needs to be studied in detail in the future.

There are certain limitations to this study, namely that we only studied the effects of short-term aeration on plant biochemistry. Different aeration times may also have inconsistent effects on plant growth. Moreover, our experiment lasted for one year, which is a short period of time compared to a long-term bamboo mulch, and long-term monitoring of soil changes to determine physiological and biochemical changes in bamboo roots could be conducted in future studies. Due to the lack of research on the application of aeration systems to alleviate soil hypoxia caused by organic mulching, many scientific and technical problems remain. Our study could serve as a representative example of this research area and generate interest in further research on the role of post-mulching aeration in various cropping systems.

## 5 Conclusions

Mulching with organic matter resulted in a decline in soil oxygen content and a reduction in shoot yield and root growth accompanied with increasing activities of anaerobic enzymes (ADH, LDH, PDC, and AlaAT). Here we innovatively propose a mechanism for improving the degradation of bamboo forest by underground pipeline aeration and find the crosstalk between hormones and *Aux/IAAs* under mulching and thus regulate the changes of enzyme and hormones indicators. Moreover, subsurface pipe aeration increased the expression of *Aux/IAAs* genes (*Aux1*, *Aux2*, *Aux3*, and *Aux4*) and IAA concentration, and reduced ABA, GA, and ETH concentrations and limited ETH synthesis enzyme activity (ACS and ACO) in the roots. The increased soil oxygen content improved root growth and shoot yield and reduced anaerobic enzyme activity, thus enhancing root resistance to organic material mulching-induced hypoxia. These findings suggest that subsurface pipe aeration helps to mitigate mulch-induced root hypoxia in bamboo and support sustainable bamboo production.

## Data availability statement

The original contributions presented in the study are included in the article/**Supplementary Materials**. Further inquiries can be directed to the corresponding author.

## Author contributions

JG and SZ conceived and designed the experiments. JG performed the experiments. JG and RG analyzed the data. JG drafted the manuscript. SZ and RG modified the paper. All authors contributed to the article and approved the submitted version.
