# Supplementary material for: Subsurface aeration mitigates organic material mulching-induced anaerobic stress via regulating hormone signaling in Phyllostachys praecox roots
Source: Front Plant Sci. 2023 Mar 1;14:1121604. doi: 10.3389/fpls.2023.1121604 (PMC10014838; doi:10.3389/fpls.2023.1121604)
Supplement: Supplementary file 1 [file DataSheet_1.pdf]

Table S1 Average monthly high and low temperatures, relative humidity and total rainfall.

Time	Average high temperature (℃)	Average low temperature (℃)	Relative humidity (%)	Total precipitation (mm)
2021/3	17	8	82.9	61.6
2021/6	30	21	85.5	117.3
2021/9	31	21	76.1	31.9
2021/12	13	2	68.4	8.9

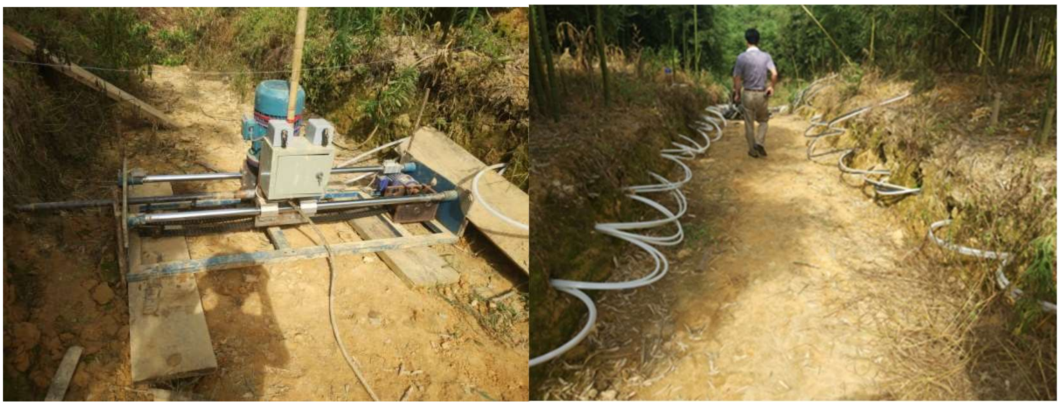

Fig S1. A picture describes the experimental arrangement.
